# Genetically engineered CXCR4-modified exosomes for delivery of miR-126 mimics to macrophages alleviate periodontitis

**DOI:** 10.1186/s12951-023-01863-w

**Published:** 2023-03-30

**Authors:** Haotian Luo, Danying Chen, Ruoyu Li, Runze Li, Yungshan Teng, Yang Cao, Xuenong Zou, Weicai Wang, Chen Zhou

**Affiliations:** 1 Hospital of Stomatology, Guanghua School of Stomatology, Guangdong Provincial Key Laboratory of Stomatology, Sun Yat-sen University, 56 Lingyuanxi Road, Guangzhou, 510055 China; 2 Guangdong Provincial Key Laboratory of Orthopaedics and Traumatology, Department of Spine Surgery, The First Affiliated Hospital, Sun Yat-sen University, Guangzhou, 510080 China

**Keywords:** Biofilm diseases, Targeted delivery, Immunomodulation, Gene therapy, Extracellular vesicles

## Abstract

Biofilm-related diseases are a group of diseases that tolerate antimicrobial chemotherapies and therefore are refractory to treatment. Periodontitis, a non-device chronic biofilm disease induced by dental plaque, can serve as an excellent *in vivo* model to study the important effects of host factors on the biofilm microenvironment. Macrophage activity is one of the key factors that modulate the progression of inflammation-driven destruction in periodontitis; therefore it is an important host immunomodulatory factor. In this study, the reduction of microRNA-126 (miR-126) with the recruitment of macrophages in periodontitis was confirmed in clinical samples, and a strategy for targeted delivery of miR-126 to macrophages was explored. Exosomes overexpressing the C-X-C motif chemokine receptor 4 (CXCR4) loaded with miR-126 (CXCR4-miR126-Exo) was successfully constructed, which reduced off-target delivery to macrophages and regulated macrophages toward the anti-inflammatory phenotype. *In vivo* local injection of CXCR4-miR126-Exo into sites of periodontitis in rats effectively reduced bone resorption and osteoclastogenesis and inhibited the progression of periodontitis. These results provide new insights for designing novel immunomodulatory factor targeted delivery systems to treat periodontitis and other biofilm-related diseases.

**Supplementary Information:**

The online version contains supplementary material available at 10.1186/s12951-023-01863-w.

## Introduction

Microbes encased by their extracellular polymeric substances (EPSs) tightly adhere to biotic or abiotic surfaces and form biofilms, resulting in persisting infections that have burdened the public health system even in developed countries [1]. Different from infections by general planktonic microbes, biofilms are difficult to be completely removed, are tolerant to antimicrobial chemotherapies, and prone to reoccur [2]. Furthermore, biofilms trigger unique host immune responses [3] which are generally ineffective, resulting in chronic inflammation. The plasticity of the macrophage polarization participates in the process of biofilm persistence; however, the detailed mechanism remains unknown [4].

Dental plaque is a mineralizing biofilm that adheres to the teeth and initiates the development of periodontitis [5, 6]. Periodontitis as the most common non-device chronic biofilm-related disease can serve as an excellent *in vivo* model to understand how host factors contribute to the biofilm microenvironment [2]. Most often, periodontitis is a chronic osteolytic disease associated with the imbalance of macrophage polarization in gingival tissue adjacent to the biofilm [7, 8]. The clinical manifestations of chronic periodontitis mainly include the irreversible destruction of tooth-supporting bony tissue and the formation of periodontal pockets [9]. Although the removal of dental calculus (mineralized biofilm) is the practical fundamental treatment for periodontitis, reconstruction of periodontal bone defects is still clinically challenging. Effective treatment methods to prevent or at least alleviate periodontal bone destruction should be explored, and macrophage polarization is one of the key immunomodulatory targets [10–12].

In periodontitis, macrophages play an important role in periodontal tissue homeostasis and defense [13], but bacteria and their products can activate macrophages to produce a large amount of pro-inflammatory factors that can cause inflammation or immune responses, and the excessive aggregation and activation of macrophages can also lead to periodontal tissue damage [14]. Periodontitis is closely related to the polarization of macrophages. Polarized macrophages play a regulatory and inducible role at different stages of periodontitis by expressing different products. During the progressive phase of inflammation, M1-type macrophages are involved in pro-inflammatory responses. They produce pro-inflammatory mediators including interleukins, which are considered to be the key factors in the progression of periodontitis and bone resorption [15]. During the quiescent phase of inflammation, M2-type macrophages proliferate massively and secrete anti-inflammatory cytokines such as interleukins. M2-type macrophages antagonize M1-type macrophage responses, modulate anti-inflammatory effects and play a role in wound healing and tissue repair [16, 17]. Therefore, M1 and M2 inter-conversions are important mechanisms by which periodontitis transits between active and inactive, and alveolar bone transits between resorption and repair. Immunomodulation of macrophages by reducing the activation of M1 macrophages or promoting their transformation to the M2 phenotype is the key to alleviating and treating chronic periodontitis.

MicroRNAs (miRNAs) regulate the plasticity and polarization of macrophages [18]. MiRNAs are short non-coding single-stranded RNA sequences that induce post-transcriptional gene silencing, thus governing many aspects of macrophage biology, including direct targeting of metabolic regulators and inflammatory signal pathways [19]. MiRNAs modulate the functional phenotypes of macrophages to counteract their pathogenicity in multiple diseases including cancer, autoimmunity, and periodontitis [18]. Aberrant expression of miRNAs has been observed to dysregulate innate and adaptive immune system cellular responses, leading to the development of periodontitis [20]. MiR-126 is one of the transcriptional regulators of periodontitis-related mediators [21–23]. In various models of inflammation, upregulation of miR-126 has been shown to be beneficial in reducing the recruitment of macrophages and inhibiting inflammation [24, 25]. Thus targeted delivery of miR-126 to macrophages may provide an effective immunomodulatory strategy for the treatment of periodontitis.

Exosomes are highly efficient cargo systems for delivering small molecules [26, 27]. Exosomes containing miRNAs can circulate systemically, transmit cellular messages, and regulate the gene expression of recipient cells [28]. Exosome-based therapeutic strategies have been reported to regulate inflammation and the immune system in rheumatoid arthritis, Sjögren’s syndrome, systemic lupus erythematosus, periodontitis, and oral squamous cell carcinoma [29]. Although several artificial nanoparticle drug delivery systems, including liposomes, have been developed, they are not very widely used due to their limited expression, instability and over-complexity [30, 31]. In comparison, exosomes have good biocompatibility, circulatory stability and low immunogenicity. More importantly, decorating the surface of an exosome with any specific essential protein is genetically easy to accomplish [32, 33]. At present, many engineering strategies are emerging that aim to improve exosomal delivery efficiency. Among those, a few studies have reported on the genetically engineered exosomes that overexpress the C-X-C chemokine receptor type 4 or CXCR4 (CXCR4-Exo) for the treatment of ulcerative colitis, tumors, and osteoporosis [34–36], demonstrating their “homing” potential towards inflammatory sites that highly express the CXCR4 ligand stromal cell-derived factor-1 (SDF-1/CXCL12). Therefore, by using this chemokine pair, CXCR4-Exo is hypothesized to improve the delivery efficiency of small functional molecules that will target macrophages involved in biofilm-related diseases such as periodontitis.

**Fig. 1:**
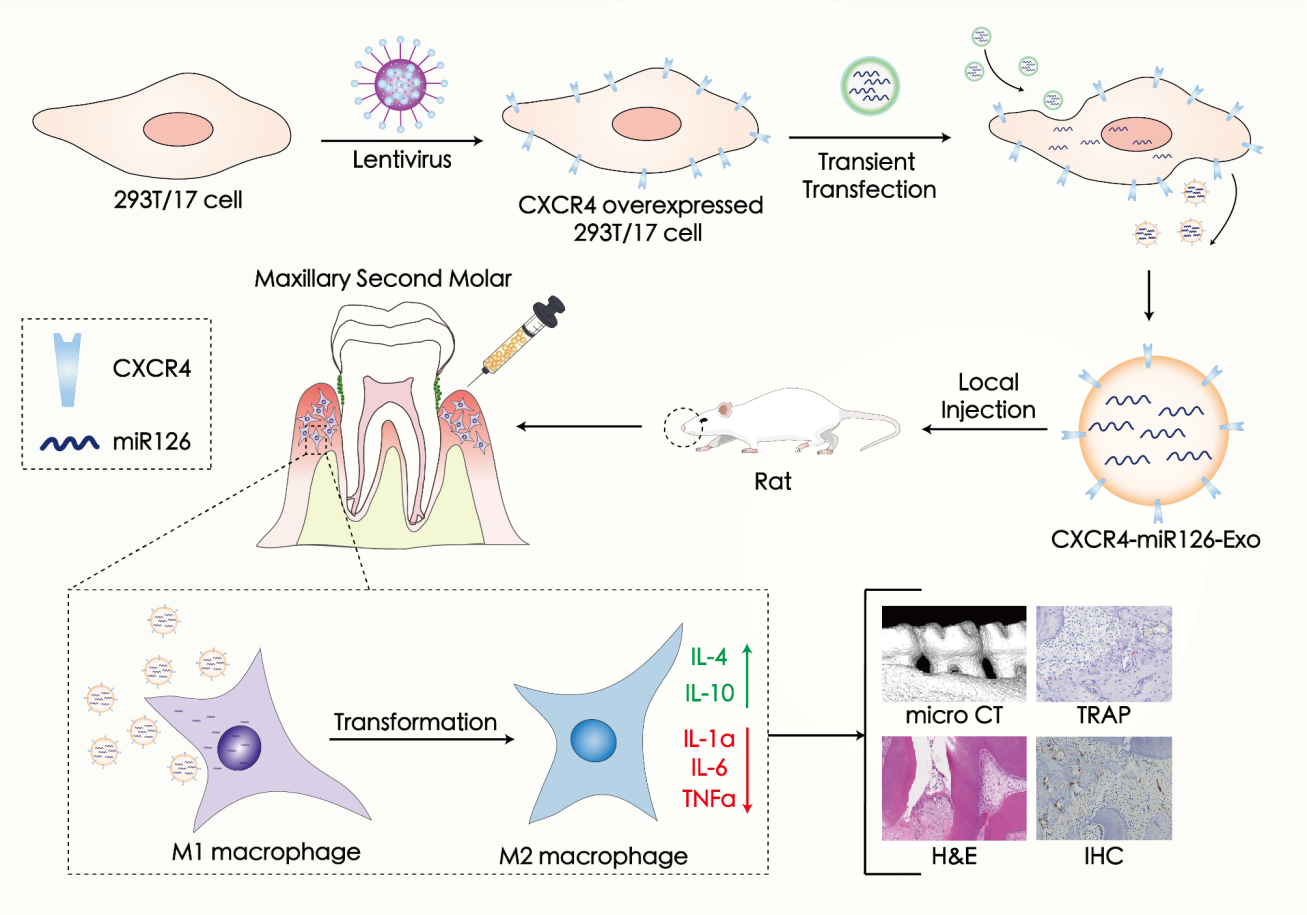
Schematic illustration of CXCR4-miR126-Exo production, targeting, and regulation of periodontitis *in vivo*

In this study, we verified the role of a small macrophage-associated anti-inflammatory molecule, miR-126, in periodontitis. In this context, we constructed an engineered CXCR4-overexpressing exosome loaded with miR-126, which efficiently delivered its cargo to macrophages, relieving periodontitis and alleviating alveolar bone loss (Fig. 1). The successful construction of the genetically engineered exosome provides a new alternative future immunomodulatory treatment for periodontitis and other biofilm-related diseases.

## Results

### miR-126 decreased with macrophage recruitment in periodontitis

**Fig. 2:**
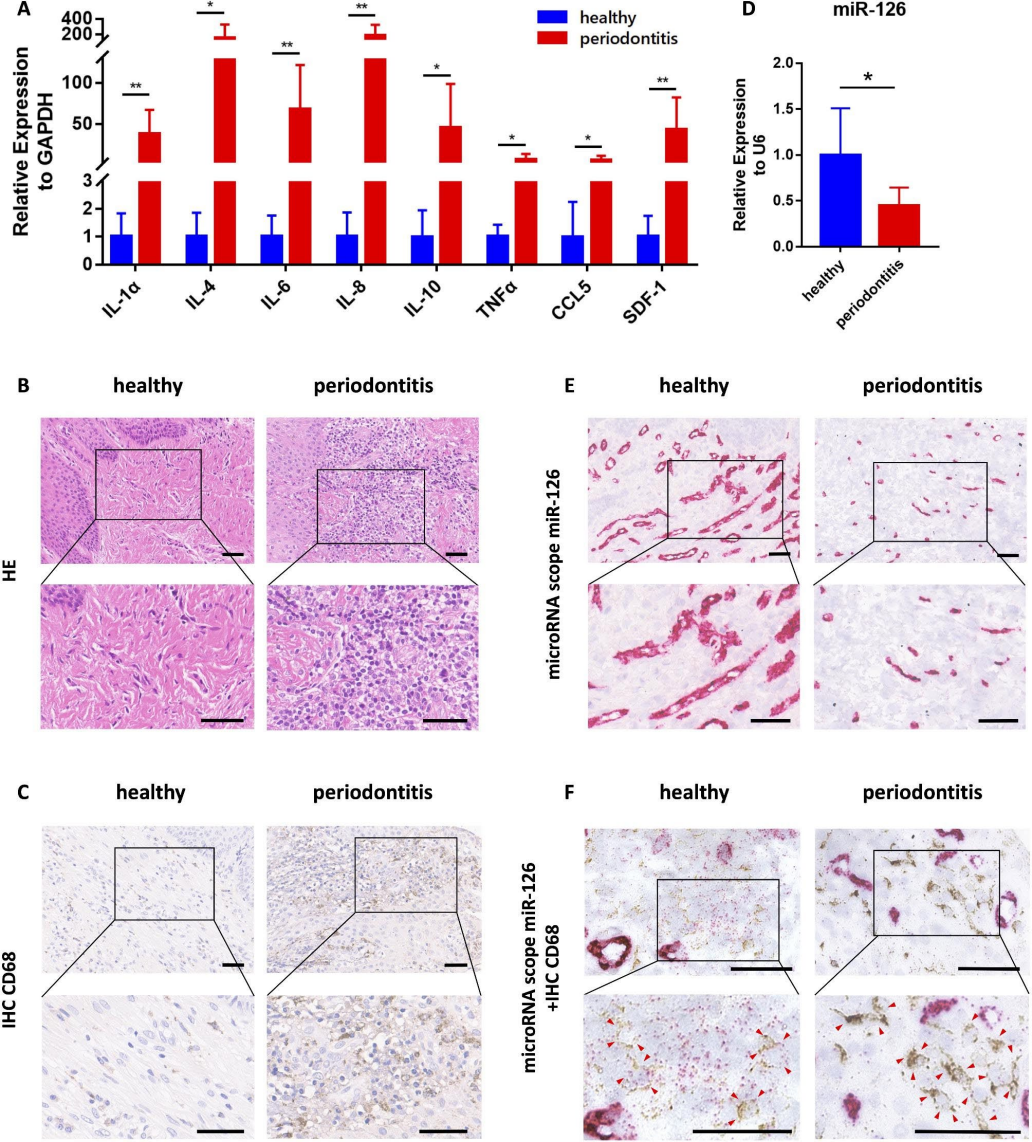
miR-126 decreased with macrophage recruitment in periodontitis **(A)** Gene expression levels of various inflammatory cytokines in the periodontal gingiva of healthy volunteers and periodontitis patients (n = 8 per group). Data are represented as mean ± SD. *P < 0.05, **P < 0.01. **(B)** H&E staining of the gingiva from healthy volunteers and periodontitis patients. Scale bar = 50 μm. **(C)** Representative IHC staining images of CD68 in two groups. Scale bar = 50 μm. **(D)** qPCR analysis of miR-126 gene expression of the periodontal gingiva from healthy volunteers and periodontitis patients (n = 8 per group). Data are represented as mean ± SD. *P < 0.05. **(E)** MicroRNA Scope staining images of miR-126 in periodontium from the two groups are shown. Scale bar = 50 μm. **(F)** MicroRNA Scope staining images of miR-126 and representative immunohistochemical staining images of CD68 are together shown. Arrows indicate CD68 positive cells. Scale bar = 50 μm.

To confirm the inflammatory conditions within periodontitis gingiva, we observed up-regulation of *SDF-1, CCL5, IL-1α, IL-4*, and other inflammatory cytokine genes by qPCR (Fig. 2A). Histological staining showed obvious infiltration of inflammatory cells (Fig. 2B) and CD68 positive macrophages were abundant (Fig. 2C and S1) in the periodontitis samples. As previous studies reported that the expression of various macrophage related microRNAs was altered in periodontitis [14–16], we assessed the expression levels of multiple periodontitis-related microRNAs by qPCR (Fig. 2D and S2) and confirmed that the levels of miR-126 were lower in periodontitis tissues compared to the healthy samples. In addition, we verified the downregulation of miR-126 in periodontitis by RNAScope (Fig. 2E and S3) with the same batch of samples as shown in Fig. 2A. When compared with the healthy tissues, miR-126 expression was significantly decreased in CD68 positive macrophages in periodontitis samples, as shown in the double-stained images (Fig. 2F and S4). We noted that miR-126 levels decreased in parallel with the recruitment of macrophages in periodontitis, suggesting that miR-126 function may be associated with macrophages.

### MiR-126 acts as an anti-inflammatory factor of M1 macrophages

**Fig. 3:**
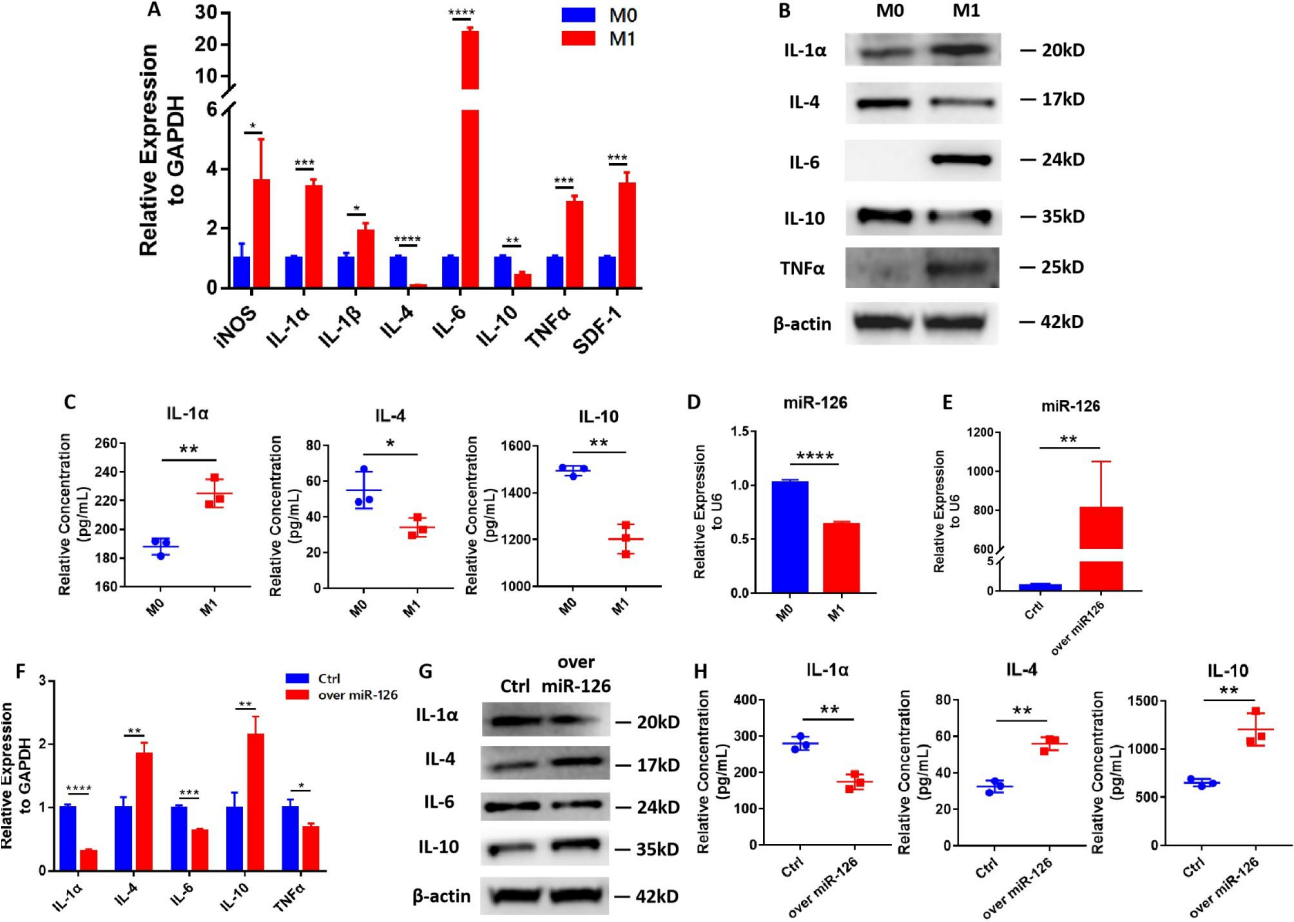
MiR-126 inhibited pro-inflammatory cytokine expression and promoted anti-inflammatory cytokine expression in M1 macrophages **(A)** The expression levels of various inflammatory cytokine genes of M0 macrophages and M1 macrophages. **(B)** Western blots showing IL-1α, IL-4, IL-6, IL-10, TNFα, and β-actin of M0 macrophages and M1 macrophages. **(C)** Detection of secreted IL-1α, IL-4, and IL-10 in M0 and M1 macrophages by ELISA. **(D)** qPCR analysis of miR-126 gene expression in M0 macrophages and M1 macrophages. **(E)** qPCR analysis to confirm the efficiency of miR-126 transfection. **(F)** qPCR analysis of inflammatory cytokine levels in M1 macrophages overexpressing miR-126 or control sequence. **(G)** Western blots showing the inflammatory cytokine protein levels of M1 macrophages overexpressing miR-126 or control sequence. **(H)** Detection of secreted IL-1α, IL-4, and IL-10 protein in M1 macrophages overexpressing miR-126 or control sequence by ELISA. Data in the bar diagrams are represented as mean ± SD. *P < 0.05, **P < 0.01, ***P < 0.001, ****P < 0.0001.

THP-1 monocyte cells were used to study the effect of miR-126 on M1 macrophages *in vitro*. M0 macrophages were polarized to M1; this was verified at the transcriptional and translational levels, showing the increased expression of iNOS and pro-inflammatory cytokines (IL-1α, IL-6, and TNFα), and decreased expression of the anti-inflammatory cytokines IL-4 and IL-10 (Fig. 3A-C). Importantly, the expression of the inflammatory chemokine *SDF-1* gene was also significantly increased in M1 macrophages (Fig. 3A).

MiR-126 was significantly decreased in M1 macrophages compared to M0 macrophages (Fig. 3D). Then, we established M1 macrophages that overexpressed miR-126 (Fig. 3E). The overexpression of miR-126 significantly attenuated the pro-inflammatory phenotype of M1 macrophages; IL-1α, IL-6, and TNFα were decreased and IL-4 and IL10 were increased at the transcriptional and translational levels (Fig. 3F-H). These findings indicate that miR-126 acts as an anti-inflammatory factor of M1 macrophages *in vitro*.

### CXCR4-miR126-Exosome generation

**Fig. 4:**
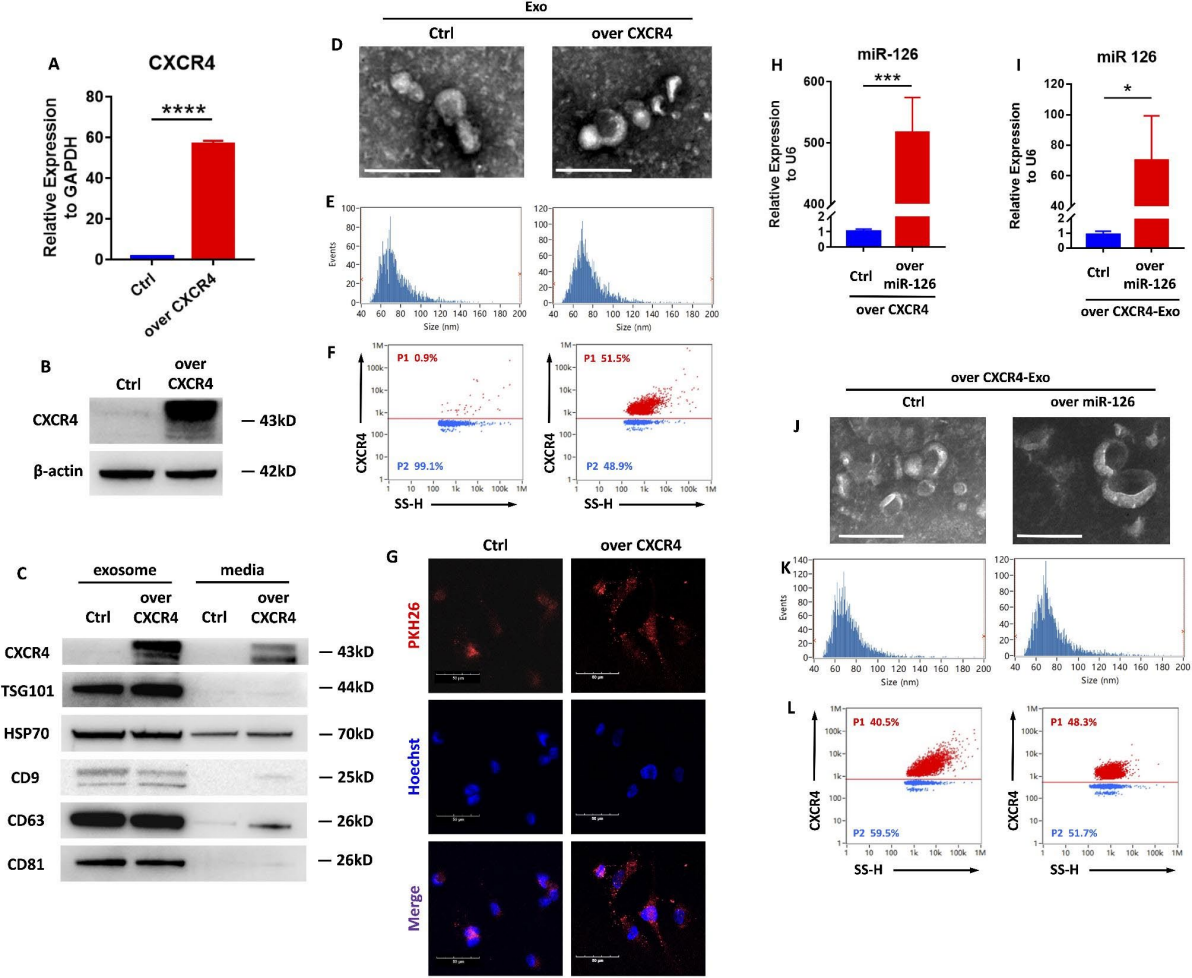
Construction of the engineered CXCR4-miR126-Exosomes **(A)** qPCR analysis of the efficiency of 293T/17 cells overexpressing CXCR4. ****P < 0.0001. **(B)** Western blots showing CXCR4 and β-actin of 293T/17 cells overexpressing CXCR4 and their controls. **(C)** Western blots showing CXCR4 and exosome markers (TSG101, HSP70, CD9, CD63, and CD81) of CXCR4-Exo and its controls compared with their conditioned media. Representative transmission electron micrographs (TEM) displaying the morphology of **(D)** CXCR4-Exo and Crtl-Exo, and **(J)** CXCR4-miR126-Exo and CXCR4-Ctrl-Exo. Scale bar = 200 nm. Particle size distribution analysis of **(E)** CXCR4-Exo and Crtl-Exo, and **(K)** CXCR4-miR126-Exo and CXCR4-Ctrl-Exo. Nano-flow cytometry analysis of CXCR4 expression on the membrane of **(F)** CXCR4-Exo and Crtl-Exo, and **(L)** CXCR4-miR126-Exo and CXCR4-Ctrl-Exo. **(G)** Representative IF images of exosome internalization from M1 macrophages co-cultured with CXCR4-Exo and Crtl-Exo. Nuclei were stained with Hoechst. Scale bar = 50 μm. **(H)** qPCR analysis of the efficiency of transfecting miR-126 into CXCR4-293T/17 cells. ***P < 0.001. **(I)** qPCR analysis of miR-126 gene expression in CXCR4-miR126-Exo and CXCR4-Ctrl-Exo. *P < 0.05. Data in the bar diagrams are represented as mean ± SD.

To construct engineered anti-inflammatory exosomes that target M1 macrophages, CXCR4-Exo was adopted to deliver miR-126 via an expected chemotactic attraction toward inflammation [37]. After transfecting 293T/17 cells with over-expressed CXCR4 lentivirus, we confirmed the significant upregulation of CXCR4 expression at the transcriptional and translational levels (Fig. 4A-B). The exosomes secreted from the CXCR4-overexpressing 293T/17 cells, CXCR4-Exo, were verified by western blots (Fig. 4C) and collected for transmission electron microscopy (TEM) and particle size analysis to test their common physical properties. We found that they were single-sided depressed spherical vesicles with a bilayer membrane structure and most were in the range of 40–120 nm in diameter (Fig. 4D-E). There was 51.5% of the CXCR4-Exo exhibited high expression levels of CXCR4 on the membrane, while only 0.9% of the Ctrl-Exo expressed CXCR4 (Fig. 4F). The uptake of CXCR4-Exo by M1 macrophages was also observed; more CXCR4-Exo were internalized than the Ctrl-Exo (Fig. 4G), however comparable uptake of the CXCR4-Exo and Ctrl-Exo by other common cell types presenting in periodontal tissues was observed (Figure S5), implying the potential of CXCR4-Exo to deliver miR-126 to M1 macrophages.

On this basis, CXCR4-miR126-Exo was further constructed. By transfecting miR-126 into the CXCR4 overexpressed 293T/17 cells successfully (Fig. 4H), CXCR4-miR126-Exo was obtained. Our qPCR analysis demonstrated that abundant miR-126 was packed in the CXCR4-miR126-Exo (Fig. 4I). TEM and particle size analyses of CXCR4-miR126-Exo showed similar results as the CXCR4-Exo (Fig. 4J-K). The expression of CXCR4 on the surface of CXCR4-Exo remained high after packing miR-126 or its negative control, accounting for 48.3% and 40.5%, respectively, implying that CXCR4 could be stably expressed on the surface of exosomes (Fig. 4L). Thus, the engineered CXCR4-miR126-Exo was correctly constructed for the delivery of miR-126 to M1 macrophages.

### CXCR4-miR126-Exo showed anti-inflammatory effects on M1 macrophages

**Fig. 5:**
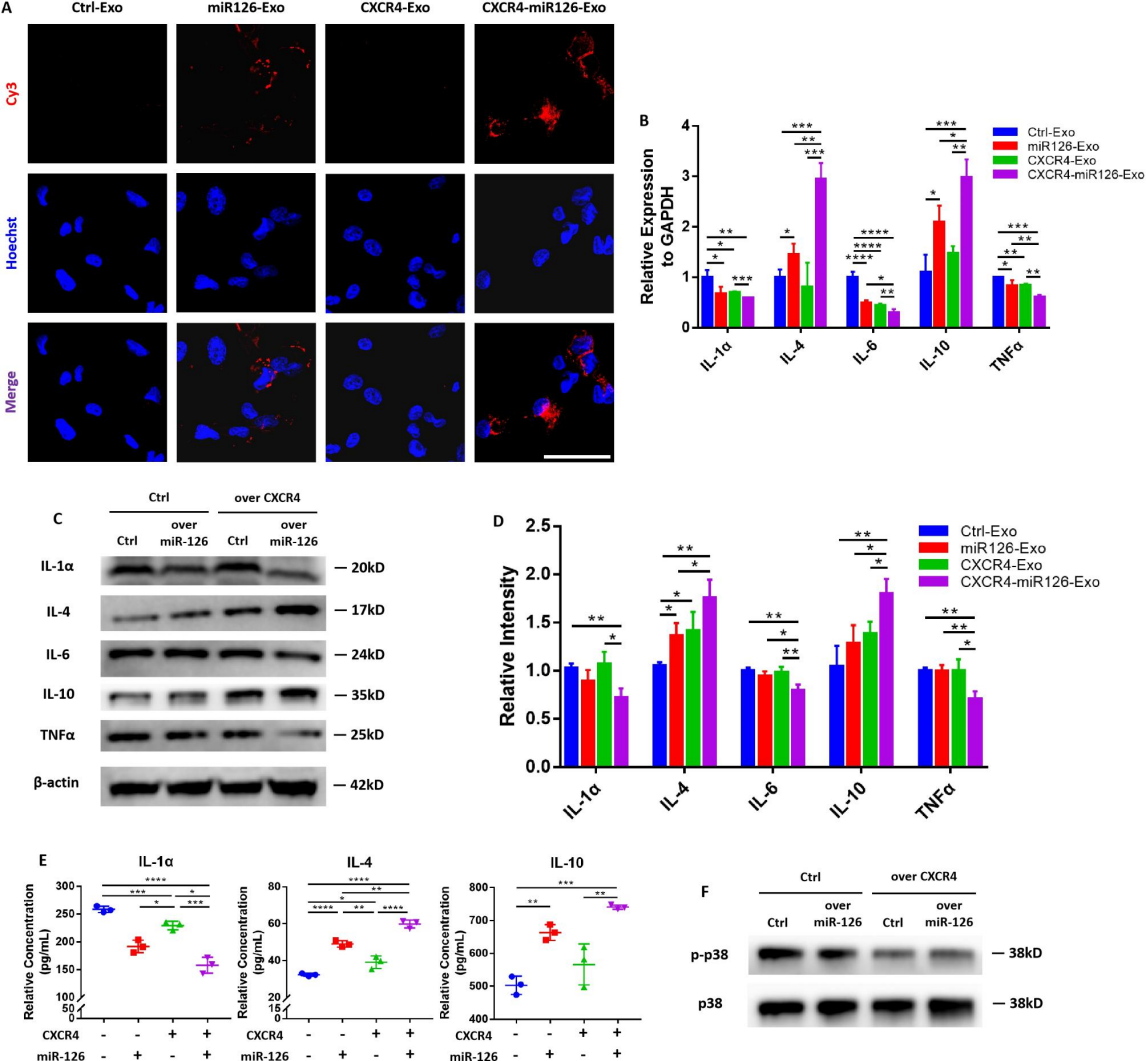
CXCR4-miR126-Exo displayed an anti-inflammatory effect on M1 macrophages **(A)** Representative immunofluorescence images of the absorption of miR-126 by M1 macrophages. MiR-126 was labeled with Cy3 fluorescence, with both Ctrl-exo and CXCR4-exo as control groups. Nuclei were stained with Hoechst. Scale bar = 50 μm. **(B)** The effect of exosomes on M1 macrophage inflammatory cytokine gene expression levels by qPCR. Cells were treated with exosomes for 48 h. **(C)** The effect of exosomes on M1 macrophage inflammatory cytokine protein levels detected by western blots, and **(D)** the relative intensity of the western blot signals. Cells were treated with exosomes for 72 h. **(E)** Detection of secreted IL-1α, IL-4, and IL-10 proteins of the exosome-treated M1 macrophages by ELISA. *P < 0.05, **P < 0.01, ***P < 0.001, ****P < 0.0001. **(F)** Representative western blots showing the p38 MAPK signal pathway proteins in the M1 macrophages of each group. Data in the bar diagrams are represented as mean ± SD.

To verify the anti-inflammatory effect of CXCR4-miR126-Exo on M1 macrophages in vitro, M1 macrophages were treated with one of four groups of exosomes: CXCR4-miR126-Exo, CXCR4-Exo, miR126-Exo, or Ctrl-Exo. MiR-126 mimics were labeled with Cy3 fluorescence and packaged in exosomes. Increased incorporation of miR-126 containing exosomes was observed in those overexpressing CXCR4 in M1 macrophages (Fig. 5A). At the transcriptional level, CXCR4-miR126-Exo showed the best anti-inflammatory effect as judged by a significant increase in *IL-4* and *IL-10* gene expression and a decrease in *IL-1α*, *IL-6*, and *TNFα* expression, proposing an improved regulatory efficiency of CXCR4-miR126-Exo compared to miR126-Exo (Fig. 5B). Correspondingly, similar changes were observed at the translational level (Fig. 5C-E). We explored a possible mechanism of downstream signaling of miR-126 and found that it may be related to the p38 mitogen-activated protein kinase (MAPK) signaling pathway, the upregulation of which leads to the persistence of periodontitis [38]. Western blot analysis confirmed that the p38 MAPK signaling was downregulated to varying degrees in the CXCR4-miR126-Exo, CXCR4-Exo and miR126-Exo groups compared to the Ctrl-Exo group (Fig. 5F). Together, these findings demonstrated the promising application of engineered CXCR4-miR126-Exo to regulate M1 macrophages in periodontitis.

### CXCR4-miR126-Exo attenuated periodontitis progression in rats

**Fig. 6:**
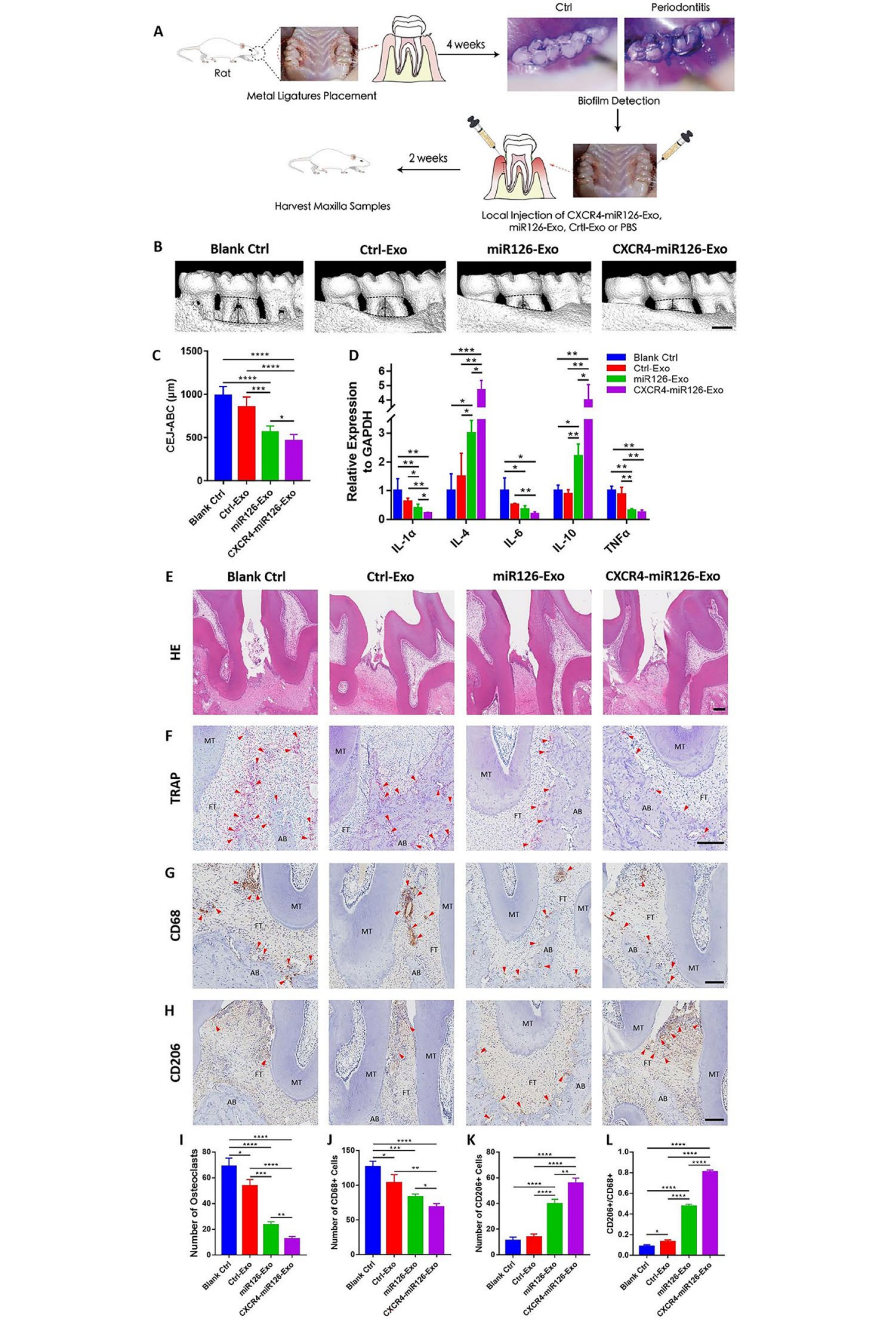
CXCR4-miR126-Exo attenuated the progression of inflammation and promoted bone repair in a rat periodontitis model **(A)** A flow diagram showing the experimental procedure for detection of biofilm and periodontal injection of exosomes in periodontitis rats. **(B)** Representative images of a 3D reconstruction of the maxillary second molar and alveolar bone of the Blank Ctrl group, the Ctrl-Exo group, the miR126-Exo group and the CXCR4-miR126-Exo group. Scale bar = 1 mm. **(C)** Statistical analysis of the distance from the cementoenamel junction (CEJ) to the alveolar bone crest (ABC) in each group. **(D)** The expression of various inflammatory cytokines in the gingiva of the rats in each group. Representative images of **(E)** H&E and **(F)** TRAP staining of the periodontium in each group. Scale bar = 200 μm. AB: alveolar bone; FT: fibrous tissues; MT: molar tissues; Arrows point to the positive area. Representative immunohistochemical staining images of **(G)** CD68 and **(H)** CD206 in each group. Scale bar = 200 μm. **(I)** Statistical analysis of the number of osteoclasts in each group as determined by TRAP staining. Statistical analysis of the IHC staining of periodontal **(J)** CD68 + cells and **(K)** CD206 + cells in each group. **(L)** Statistical analysis of the ratio of CD206 + cells/CD68 + cells from the IHC staining. Data in the bar diagrams are represented as mean ± SD. *P < 0.05, **P < 0.01, ***P < 0.001, ****P < 0.0001.

To further confirm the ability of CXCR4-miR126-Exo to treat periodontitis, a rat maxillary second molar periodontitis model was established. More biofilm habitats were observed around the ligation. Then, the engineered exosomes were injected into six sites around the maxillary second molar (Fig. 6A). As shown in the three-dimensional reconstruction of the four conditions (Fig. 6B-C and S6), alveolar bone resorption was most significantly attenuated by the CXCR4-miR126-Exo. Expression of inflammatory cytokine genes in periodontal gingiva tissues showed that the CXCR4-miR126-Exo group had the lowest levels of pro-inflammatory cytokines and the highest levels of anti-inflammatory cytokines (Fig. 6D). Hematoxylin & Eosin (H & E) staining showed various degrees of damage to the gingival sulcus epithelium of each group, with the CXCR4-miR126-Exo group showing the least damage and the Blank Ctrl group the most damage (Fig. 6E). Tartrate-resistant acid phosphatase (TRAP) staining revealed that a small number of osteoclasts were distributed in the CXCR4-miR126-Exo group and the miR126-Exo group, while the Ctrl-Exo group and the Blank Ctrl group presented a large number of osteoclasts, suggesting that CXCR4-miR126-Exo inhibited osteoclast activation thereby reducing alveolar bone loss (Fig. 6F&I). In addition, immunohistochemical staining demonstrated that the CXCR4-miR126-Exo reduced the aggregation of CD68 + cells and concurrently induced a significant increase in the number of CD206 + cells. Few CD206 + cells were observed in both control groups (Fig. 6G-H&J-K and S7). The ratio of CD206+/CD68 + was ~ 0.8 in the CXCR4-miR126-Exo group, implying that most macrophages were of the M2 anti-inflammatory phenotype. In contrast, the miR-126 group had a ratio of ~ 0.5; ratios in both control groups were < 0.2 (Fig. 6L). These findings demonstrate the protective effect of CXCR4-miR126-Exo on periodontal tissues.

## Discussion

The immunomodulation of biofilm related host response in combination with anti-microbial therapy is sometimes necessary to reduce tissue damage, especially in situations where the elimination of biofilm is not feasible. In this study, we demonstrated the construction of a genetically engineered exosome capable of targeting macrophages to deliver miR-126 as a feasible immunomodulatory therapy to relieve periodontitis in rats. We found that this inhibited the macrophage inflammatory response and reduced bone loss.

Macrophages offer a feasible target for the immunomodulation of periodontitis. Macrophages exhibit different responses to different pathogenic stimuli and are one of the key cells involved in chronic inflammation-related pathologies [39]. In periodontitis, macrophages play an important role in periodontal tissue damage and repair. The transition from the M1 to M2 phenotype is an important mechanism by which periodontitis converts from progression to quiescence, and is beneficial for tissue repair [40]. Several studies have attempted to modulate the periodontitis immune microenvironment by modulating M1 macrophages. At the post-transcriptional level, some exogenous biological macromolecules were shown to affect the progression of periodontitis [41–43]. However, post-transcriptional intervention may risk poor biocompatibility, and the immune system may simply remove the administered exogenous macromolecules, thus reducing the efficiency of the intervention. At the transcriptional level, gene therapy has taken advantage of high transfection efficiencies and low mutation rates to become the most promising treatment strategy for periodontitis [44]. MicroRNAs (miRNAs) are one of the candidate tools that can be used to regulate gene expression due to their small molecule size and their diverse functions [45, 46]. Increasingly, studies have identified multiple miRNAs that regulate the plasticity and polarization of macrophages [18]. MiRNAs not only govern many aspects of macrophage biology, such as direct targeting of metabolic regulators and inflammatory pathways [19] but also regulate the functional macrophage phenotypes. Therefore, miRNAs modulation of macrophages deserves closer exploration.

MiR-126 expression is downregulated in inflammatory diseases [24, 25] and has been detected in human periodontal diseased gingival tissues, which differs from healthy gingival tissues [47]. Overexpression of MiR-126 decreased the expression of interleukin (IL)-1β [48], IL-6 and TNFα [49], and these studies were consistent with the results of our experiments. These inflammatory cytokines are important inducers of inflammation mediated by mitogen-activated protein kinase (MAPK) and nuclear factor-kappa B (NF-κB), and activation of these signaling pathways can lead to the development of periodontitis [38]. The results of this study confirmed the direct target of miR-126, which alleviated periodontitis by inhibiting the activation of the MAPK pathway. In vitro, we further verified a marked decrease of miR-126 in M1 macrophages. We also explored the function of miR-126 on M1 macrophages by using miR-126 overexpression in M1 macrophages, resulting in reduced expression of key pro-inflammatory cytokines [9] and polarization to the M2 phenotype. However, other miRNAs and their functions have yet to be further clarified in macrophages. Although the present study may have been limited by only focusing on one miRNA, this study is the first to show that targeted delivery of miR-126 to macrophages can play an immunomodulatory role in periodontitis.

As shown by our study, overexpression of miR-126 successfully promoted the transformation of M1-type macrophages to M2-type. However, we needed a carrier for targeted delivery of miR-126 to macrophages. As natural carriers of signaling molecules, exosomes offer a set of attractive properties for therapeutic delivery, including excellent biocompatibility, circulation stability, biological barrier permeability, low immunogenicity, and low toxicity [33, 50]. However, biodistribution characteristics of naturally secreted exosomes show limited attraction towards specific cell types, thus requiring detailed engineering strategies for targeted delivery [51]. Most of these strategies focus on remodeling the exosomal surface, including expressing chemokine receptors [52] or coating with antibody fragments [53]. SDF-1, also known as CXC chemokine CXCL12, is an important chemokine in the inflammatory process because it broadly recruits leukocytes that express its specific ligand, CXCR4 [54]. SDF-1 is significantly upregulated in periodontitis [55] and in M1 macrophages, which was verified in our study. Exosomes with enriched surface CXCR4, CXCR4-Exo, were reported in several studies to be useful for treating some inflammatory diseases [34–36], and have proven their potential in the targeted-delivery of miRNA to macrophages. The detailed mechanisms of the chemotaxis-like function of the CXCR4-Exo remain obscure. It is speculated that CXCR4-Exo delivers the CXCR4 overexpressing lentivirus, CXCR4 mRNAs, and CXCR4 proteins together with miRNAs to the targeted cells and this relates to the improved delivery efficiency [56]. Consistent with previous research, our study employed human embryonic kidney cells (293T/17 cells) as the exosome supplier to construct the CXCR4-Exo after lentiviral transfection, confirming the increased internalization of exosomes with enriched CXCR4 by M1 macrophages in vitro.

We constructed the CXCR4-miR126-Exo by loading the CXCR4-Exo with miR-126. We used 293T/17 cells as exosome-derived donor cells, with the characteristic of unlimited proliferation, which was beneficial for us to obtain a large number of exosomes as a transport vehicle. However, taking into account that the ligand of CXCR4, SDF-1, is also highly expressed in bone marrow and other inflammatory tissues [57, 58], which limited the systemic targeting of CXCR4-Exo, we chose to deliver the engineered exosomes by local, and not systemic, injection into the experimental rat model. The locally injected exosomes are not transported through the circulation, thus reducing drug waste, improving treatment efficiency, and minimizing the risk of adverse reactions and complications. In addition, local injection allows for better control over the extent and duration of treatment. In conclusion, our *in vivo* experiments have demonstrated that the CXCR4-miR126-Exo alleviated periodontal inflammation.

## Materials and methods

### Human samples

Samples were collected from patients at the Department of Oral and Maxillofacial Surgery, Hospital of Stomatology, Sun Yat-sen University, Guangzhou, China. After obtaining informed consent from the patients, gingival tissues were collected during the surgery performed on patients with chronic periodontitis who required the extraction of loose teeth or healthy participants who needed to extract their third molars. The samples were divided into an experimental group (patients with chronic periodontitis) and a control group (systemically healthy patients). The inclusion criteria for the experimental group were: (1) Patients aged 18–80 who were newly diagnosed with periodontitis based on the 2017 classification stages III and IV determined using clinical attachment loss (CAL) and radiographic bone loss for the diagnosis of periodontitis [59]; (2) No systemic treatment within the last 6 months for chronic periodontal diseases such as subgingival scaling, supragingival scaling, or those on anti-inflammatory drugs; (3) No systemic disease (e.g., cancer, kidney or liver failure, or heart disease), other acute or chronic inflammatory diseases, or mental illness. (4) Able to sign the informed consent form. Subjects with one of the following conditions were not included in the trial: (1) Age under 18 years old or over 80 years old; (2) Patients with other systemic diseases, acute or chronic inflammatory diseases, or mental illnesses; (3) Pregnant or lactating women; (4) Systemic treatment for chronic periodontal disease within the last 6 months. The inclusion criteria for the control group were as follows: (1) Healthy subjects over 18 years old (including those who were 18 years old); (2) Gender: male or non-pregnant, non-lactating female; (3) The weight of male subjects was not less than 50 kg, the weight of female subjects was not less than 45 kg, and the body mass index (BMI) of the subjects was between 18.0 and 28.0 kg/m^2^, where BMI = weight (kg)/height^2^ (m^2^), including the critical value; (4) No history of major diseases and drug allergies; (5) No smoking or long-term alcohol consumption; (6) Clinical and imaging examination confirmed the healthy periodontal condition and no inflammatory disease. Participants were recruited according to a pre-determined minimum sample size using *a priori* assessment using a PPD index estimate based on type I error = 0.05, power = 0.80, and effect size (ES) f = 0.25.

### Quantitative real-time PCR

RNAzol reagent (Genecopoeia, USA) was used to extract total RNA from the samples, and the PrimeScript RT Master Mix (Takara, Japan) was used for reverse transcription. Quantitative real-time PCR was performed with an ABI QuantStudio5 (USA) and the AceQ qPCR SYBR Green Master Mix (Vazyme, China). cDNA of the microRNA was generated using a miRNA cDNA Synthesis Kit (CoWin Biosciences, China), and gene expression was measured by qPCR with a miRNA qPCR Assay Kit (CoWin Biosciences, China). *GAPDH* was used as the internal control of the total RNA, and *U6* was the control for microRNA. The primer sequences are shown in Table S1. The relative quantity of genes was calculated using the 2^−ΔΔCt^ method.

### Immunohistochemistry (IHC)

The animal samples were decalcified in ethylene diamine tetra-acetic acid (EDTA, Servicebio, China) for 4 weeks after microcomputed tomography analysis. The samples were dehydrated by a graded series of alcohol, embedded in paraffin, and cut into 6-µm sections for staining. The sections were stained with hematoxylin & eosin (H&E) and tartrate-resistant acid phosphatase (TRAP, Servicebio, China) according to the manufacturer’s protocol. For immunohistochemistry, the sections were dewaxed, rehydrated, and antigen retrieval was conducted before being incubated with the anti-CD68 or the anti-CD206 (1:200, Servicebio, China) primary antibodies overnight at 4℃, then incubated with the secondary antibodies for 1 h at room temperature. Diaminobenzidine (DAB, Servicebio, China) was used for visualization, followed by counterstaining with hematoxylin (Servicebio, China). Non-specific IgG was used as a negative control. Then the sample sections were imaged with an Aperio AT2 slide scanner (Leica Biosystems, Germany).

### MicroRNA scope

To localize miR-126 expression in the tissue sections, a miRNAScope™ HD (RED) Assay Kit (Bio-techne, USA) was used according to the manufacturer’s instructions and established protocols. Briefly, after dewaxing, hydrogen peroxide blocking, and heat-mediated antigen retrieval using a steamer, Protease III was applied. After the hybridization probe and Hybridize Amp1-6 were added dropwise in sequence, diaminobenzidine (DAB, Servicebio, China) was used for visualization of miR-126, and the slides were then counterstained and mounted. Scrambled miRNA was used as a negative control.

### Cell culture

293T/17 cells [purchased from Procell Life Science&Technology Co., Ltd (China)] were cultured in Dulbecco’s Modified Eagle Medium, high glucose, with Sodium Pyruvate (Biosharp, China) containing 10% fetal bovine serum (FBS, BioInd, Israel) at 37℃ in a 5% CO_2_ humidified cell culture incubator.

THP-1 cells [purchased from Shanghai Zhong Qiao Xin Zhou Biotechnology Co., Ltd (China)] were cultured in Roswell Park Memorial Institute Medium (RPMI)-1640 (Gibco, USA) containing 10% fetal bovine serum (FBS, Gibco, USA), 1% v/v penicillin/streptomycin (Gibco, USA) and 1% v/v GlutaMAX (Gibco, USA). To obtain M0 macrophages, THP-1 cells were treated with 10 mg/mL of Phorbol 12-myristate 13-acetate (PMA, Solarbio, China) for 24 h. To induce M0 macrophage polarization into M1 macrophages, M0 macrophages were further treated with 1 µg/mL LPS (Sigma, USA) and 50 ng/mL IFN-γ (Peprotech, USA) for 24 h (Figure S8).

### MicroRNA transfection

MiR-126 mimics from two species (miR-126 [*Homo sapiens*] (Gene ID: 406913) and miR-126 [*Rattus norvegicus*] (Gene ID: 100314237)) and their negative controls were purchased from Genepharma, China. MiR-126 mimics (Genepharma, China) and negative control (Genepharma, China) were transfected into THP-1 cells and 29T3/17 cells by the RFect siRNA/miRNA Transfection Reagent (Bio-generating Biotechnology, China) according to the manufacturer’s instructions. After transfection for 24 h, cells were cultured for a further 48 h. According to preliminary experiments performed, the final concentrations of transfected miRNAs were 30 nM (miR-126 mimics) and 30 nM (negative control) (Figure S9), and the efficiency of transfection was verified by qRT-PCR.

### Western blot

RIPA Lysis Buffer (Strong, CoWin Biosciences, China) with 1% Protease Inhibitor Cocktail (CoWin Biosciences, China) was used to extract proteins from M1 macrophages. The concentration of total protein was measured using a Bicinchoninic Acid Protein Assay Kit (BCA, CoWin Biosciences, China). SDS-PAGE Loading Buffer and the protein samples were mixed and heated in a boiling water bath for 3–5 min. Then, the proteins were resolved by SDS-PAGE (Genscript, China) and transferred to a polyvinylidene fluoride (PVDF) membrane (Millipore, USA) with a wet transfer blotting system (Bio-Rad, China). The membranes were blocked with TBS-Tween (CoWin Biosciences, China) plus 5% bovine serum albumin for 1 h, then were incubated at 4℃ overnight with primary antibodies: Polyclonal anti-IL 1α (1:500, Affinity Biosciences, China), Polyclonal anti-IL 4 (1:500, Affinity Biosciences, China), Polyclonal anti-IL 6 (1:500, ZENBio, China), Polyclonal anti-IL 10 (1:500, Affinity Biosciences, China), Polyclonal anti-TNFα (1:500, Affinity Biosciences, China), polyclonal rabbit anti-β-Actin (1:1000, Servicebio, China), monoclonal rabbit anti-Phospho-p38 MAPK (1:1000, CST, USA), and monoclonal rabbit anti-p38 MAPK (1:1000, CST, USA), then incubated with secondary antibodies (1:10000) for 1 h at room temperature. Signals were detected with the ECL western blotting substrate kit (Millipore, USA), and the relative intensity of every immunoreactive protein band was quantified with the ImageJ software (NIH, USA, 1.8.0).

### ELISA

The concentrations of IL-1α, IL-4 and IL-10 in cell-culture supernatant were detected using a commercially available ELISA kit (MMbio, China) according to the manufacturer’s instructions and established protocols. Briefly, the ELISA reagents were added to the samples and incubated at 37℃ for 1 h. After washing the wells 5 times with the wash solution, the chromogenic reagent was added and incubated at 37℃ in the dark for 15 min. The reaction was stopped using the stop solution. The absorbance (OD value) was measured at a wavelength of 450 nm using a microplate reader.

### Preparation of the CXCR4-overexpressing 29T3/17 cells

293T/17 cells were cultured as described above. A PCR-amplified gene fragment encoding human *CXCR4* (Gene ID: 7852) or rat *Cxcr4* (Gene ID: 60628) was cloned into a lentiviral vector (Genepharma, China). 293T/17 cells (1.5 × 10^6^) were seeded in 6-cm culture dishes for 12 h and then transfected with the CXCR4 recombinant lentivirus or the negative control at a multiplicity of infection (MOI) of 50 for 24 h. In the next 5 days, lentivirus-free medium with 2 µg/mL Puromycin dihydrochloride (Puro, APExBIO, USA) was used for the selection of transfected cells, and the transfected cells were expanded for further experiments. The transfection efficiency of CXCR4 in the 293T/17 cells was detected by the GFP fluorescence (Figure S10).

### Exosome isolation and characterization

For exosome collection, 293T/17 cells were allowed to reach 80% confluence in DMEM basal medium without FBS for 48 h, after which, the supernatant was collected and centrifuged at 300×g for 10 min at 4℃ to remove any cells or large cellular fragments. Differential centrifugation was used to isolate the exosomes: the supernatant was first centrifuged at 2000×g for 20 min at 4℃ then 10,000×g for 30 min at 4℃ to remove microvesicles, and filtered through a 0.22 μm filter. The samples were carefully transferred to ultracentrifuge tubes (Beckman Coulter, USA) and centrifuged at 110,000×g for 70 min at 4℃. The supernatant was removed, leaving a residual volume of 1 mL containing the exosome pellet in the tubes. After replenishment with filtered PBS, the ultracentrifuge tubes were centrifuged again at 110,000×g for 70 min at 4℃. The exosomes were reconstituted in filtered PBS and stored at -80℃.

A micro BCA Protein Assay Kit (CoWin Biosciences, China) was used to measure the concentration of exosomes. Western blotting was conducted to confirm the presence of exosome markers such as CD9, CD63, CD81, TSG101 and HSP70. The exosomes were added drop-wise on a copper grid and stained with 2% (w/v) phosphotungstic acid for 5 min. Then the morphology of the exosomes was observed with a transmission electron microscope (TEM, Tecnai Spirit, FEI, USA). The exosomes were stained with 10µL Allophycocyanin- (APC) labeled anti-human CXCR4 Antibody (Biolegend, USA) in the dark for 20 min, then the particle size distribution and CXCR4 expression on the exosome membrane were examined by Flow NanoAnalyzer (Nanofcm, China).

### Exosome labelling and internalization

A PKH26 red fluorescent dye kit (Umibio, China) was used to label the exosomes. Purified exosomes were incubated in 50µL PKH26 for 10 min at 37℃, washed, and centrifuged at 110,000×g for 70 min at 4℃ to remove unbound dye. Labeled exosomes (10 µg for every 10^5^ cells) were added to the M1 macrophages, human bone marrow mesenchymal stem cells (HBMSC), human gingival epithelial cells (HGEC) and human umbilical vein endothelial cells (HUVEC) seeded on glass slides and incubated at 37℃ for 12 h. Next, the cells were fixed in 4% paraformaldehyde (PFA) for 15 min and permeabilized in 0.1% Triton-X in PBS for 15 min. After washing twice with PBS, the samples were stained with Hoechst 33,342 (Beyotime Biotechnology, China) for 15 min. The uptake of exosomes by different cells was observed and imaged by using a laser scanning confocal microscope (Olympus, Japan).

### Animal samples

Eight-week-old male Sprague-Dawley (SD) rats weighing 300–400 g were purchased from Sun Yat-sen University. The SD rats were randomly divided into four groups: (1) CXCR4-miR126-Exo; (2) miR126-Exo; (3) Ctrl-Exo; and (4) Blank Ctrl. An experimental periodontitis model in rats that employed the ligation of molars was frequently used in dental research to investigate the pathology of periodontitis. Ligatures around the cervical area of the molars in rats allowed for plaque accumulation, periodontal inflammation and alveolar bone loss in the periodontal tissues, similar to the effects observed in human periodontitis [60–63]. The periodontitis models were established in SD rats as previously described [64], with modifications to ensure successful model construction (Figure S11). Briefly, the rats were anesthetized with 1% pentobarbital (0.4 mL/100 g) via intraperitoneal injection. A 1 mm metal ligature was wrapped around the neck of the bilateral maxillary second molars for 4 weeks to establish chronic periodontitis. Plaque Disclosing Solution (Miradent, Germany) was used to detect biofilm formation around the ligature. The solution was applied around the rat molars and gently rinsed with physiological saline after five minutes. The purple coloring of the teeth indicated plaque biofilm. For exosome treatment, the ligature was removed and exosomes (300 µg) from each group or PBS were injected into the periodontium of the second maxillary molar of each rat. After 2 weeks, the rats were euthanized to harvest their maxillae and gingiva tissues. The samples were fixed in 4% paraformaldehyde solution for 24 h washed three times in PBS and then stored in 75% ethanol for subsequent analysis.

### Micro-CT

The maxillae of SD rats were placed in sample containers and scanned by a micro-CT system (Scanco Medical µCT 50, Switzerland), with the voltage set at 70 kV and electric current at 114µA. The three-dimensional microstructure was reconstructed and analyzed using the RadiAnt dicom Viewer (Poland). The distance between the cementoenamel junction and the alveolar bone crest (CEJ-ABC distance) was measured at six sites around the maxillary second molars.

### Statistical analysis

All data are presented as mean ± standard deviation (SD). At least three independent experiments were performed. The GraphPad Prism software (USA) was used for the statistical analyses. Comparisons between two groups were performed using independent unpaired two-tailed Student’s *t*-test, and comparisons between more than two groups were performed using one-way analysis of variance (ANOVA). *P* values < 0.05 were considered to indicate statistical significance.

## Conclusion

Macrophage is a good target for immunomodulatory therapy to treat periodontitis. We showed proof of this concept by using an engineered CXCR4-miR126-Exo. The delivery of miR-126 to M1 macrophages effectively inhibited the expression of pro-inflammatory cytokines and promoted the expression of anti-inflammatory cytokines, thereby polarizing M1 macrophages into an M2 phenotype and attenuating inflammatory tissue damage in the periodontitis rat model. The CXCR4-Exo targeted-delivery of miRNAs to macrophages also provides new insights for the development of therapeutic approaches to treat other biofilm-related diseases.

## Electronic supplementary material

Below is the link to the electronic supplementary material.

Supplementary Material 1

## Data Availability

The data that support the findings of this study are available from the corresponding author upon reasonable request.
