# Supplementary material for: Genetically engineered CXCR4-modified exosomes for delivery of miR-126 mimics to macrophages alleviate periodontitis
Source: J Nanobiotechnology. 2023 Mar 30;21:116. doi: 10.1186/s12951-023-01863-w (PMC10061745; doi:10.1186/s12951-023-01863-w)
Supplement: Supplementary file 1 — Supplementary Material 1 [file 12951_2023_1863_MOESM1_ESM.pdf]

Supplementary materials

Table S1 The qPCR Primer Sequences

Gene	Primer	Sequences (5'-3')
Homo sapiens		
IL-1α	Forward	TGTATGTGACTGCCCAAGATGAAG
	Reverse	AGAGGAGGTTGGTCTCACTACC
IL-1β	Forward	CCACAGACCTTCCAGGAGAATG
	Reverse	GTGCAGTTCAGTGATCGTACAGG
IL-4	Forward	CCGTAACAGACATCTTTGCTGCC
	Reverse	GAGTGTCCTTCTCATGGTGGCT
IL-6	Forward	AGACAGCCACTCACCTCTTCAG
	Reverse	TTCTGCCAGTGCCTCTTTGCTG
IL-8	Forward	GAGAGTGATTGAGAGTGGACCAC
	Reverse	CACAACCCTCTGCACCCAGTTT
IL-10	Forward	TCTCCGAGATGCCTTCAGCAGA
	Reverse	TCAGACAAGGCTTGGCAACCCA
TNFα	Forward	CTCTTCTGCCTGCTGCACTTTG
	Reverse	ATGGGCTACAGGCTTGTCACTC
CCL5	Forward	TACACCAGTGGCAAGTGCTC
	Reverse	TGTACTCCCGAACCCATTTC
SDF1	Forward	CTCAACACTCCAAACTGTGCCC
	Reverse	CTCCAGGTACTCCTGAATCCAC
iNOS	Forward	GCTCTACACCTCCAATGTGACC
	Reverse	CTGCCGAGATTTGAGCCTCATG
CXCR4	Forward	CTCCTCTTTGTCATCACGCTTCC
	Reverse	GGATGAGGACACTGCTGTAGAG
GAPDH	Forward	GTCTCCTCTGACTTCAACAGCG
	Reverse	ACCACCCTGTTGCTGTAGCCAA
miR-126	Forward	GCGTCGTACCGTGAGTAAT
	Reverse	GCAGGGTCCGAGGTATTC
miR-130a	Forward	CACATTGTGCTACTGTCT
	Reverse	GAACATGTCTGCGTATCTC
miR-142-3p	Forward	GGGGGTGTAGTGTTTCCTA
	Reverse	CAGTGCGTGTCGTGGA
miR-146a	Forward	GAGAACTGAATTCCATGG
	Reverse	GAACATGTCTGCGTATCTC
miR-155	Forward	TGCTAATCGTGATAGGGG
	Reverse	GAACATGTCTGCGTATCTC
miR-223	Forward	CGTGTATTTGACAAGCTG
	Reverse	GAACATGTCTGCGTATCTC
U6	Forward	CTCGCTTCGGCAGCACA
	Reverse	TTTGCGTGTCATCCTTGCG
Rattus norvegicus		
IL-1α	Forward	CGCTTGAGTCGGCAAAGAAA
	Reverse	AGACAGATGGTCAATGGCAGA
IL-4	Forward	CAAGTCTGGGGTTCTCGGTG
	Reverse	AGACCGCTGACACCTCTACA
IL-6	Forward	GTCAACTCCATCTGCCCTTCA
	Reverse	GAAGGCAACTGGCTGGAAGT
IL-10	Forward	ACTTCGATCCTAAGGCTGGC
	Reverse	GCAAGCGAAAGGACACCATT
TNFα	Forward	GGAGGGAGAACAGCAACTCC
	Reverse	GCCAGTGTATGAGAGGGACG
GAPDH	Forward	GCAAGTTCAACGGCACAG
	Reverse	GCCAGTAGACTCCACGACAT

Figure S1:

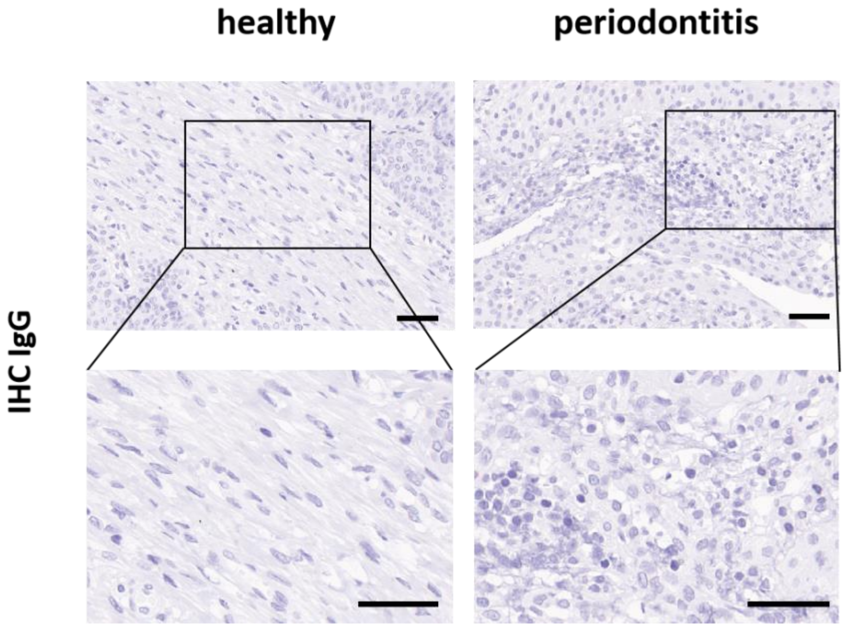

Figure S1 Representative immunohistochemical staining images of IgG as a negative control in two groups. Scale bar=50μm.

Figure S2:

The results showed that there were no significant differences in other periodontitis-related microRNAs between the two groups were detected.

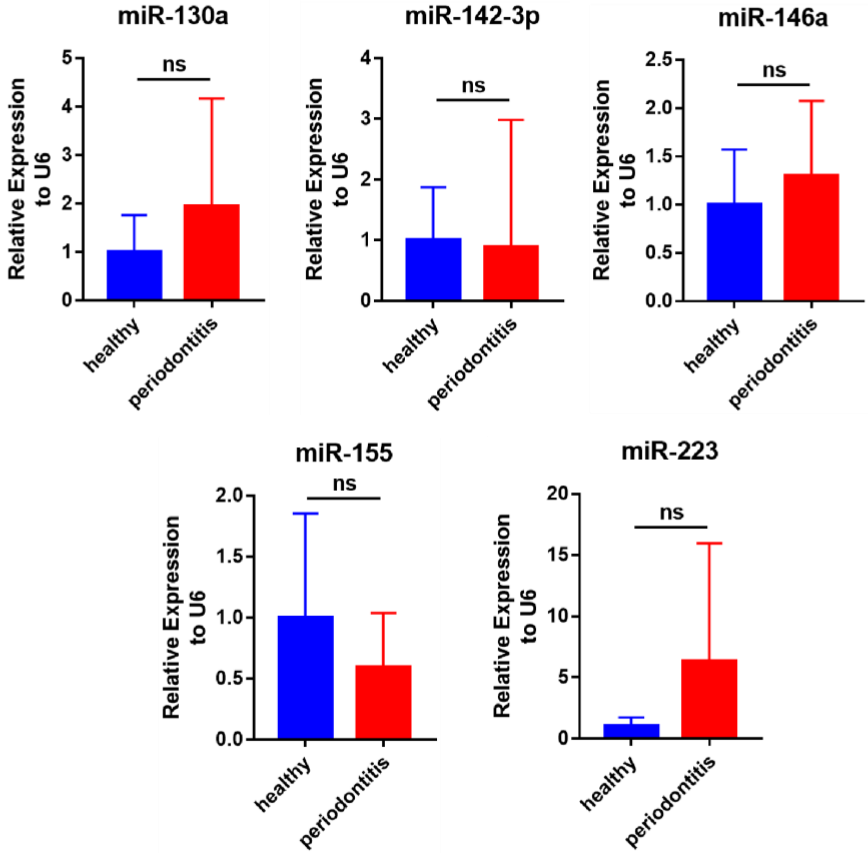

Figure S2 QPCR analysis of miR-130a, miR-142-3p, miR-146a, miR-155 and miR-233 gene expression of the periodontal gingiva from healthy volunteers and periodontitis patients. Data are represented as mean ± SD.

Figure S3:

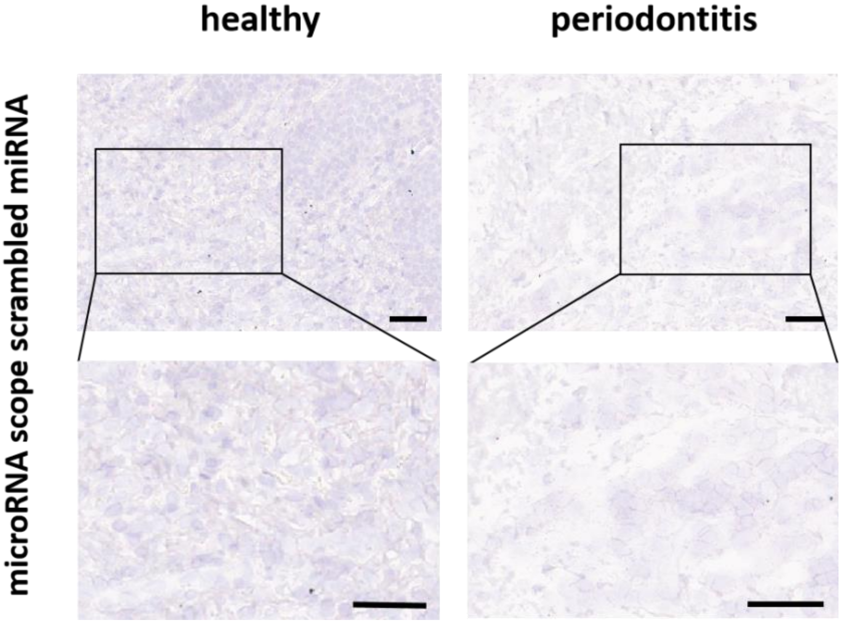

Figure S3 MicroRNA scope staining images of scrambled miRNA as a negative control in periodontium from two groups are shown. Scale bar=50μm.

Figure S4:

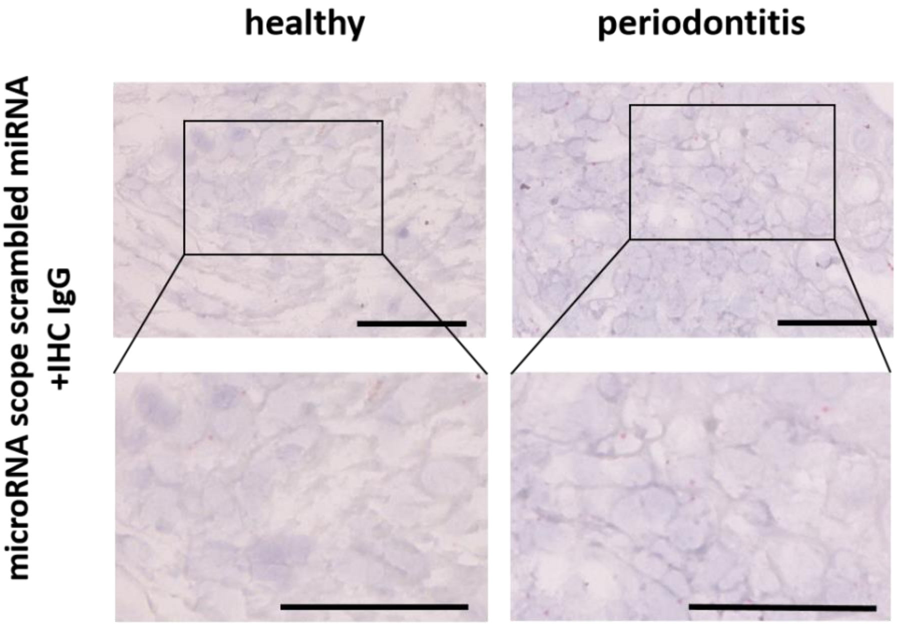

Figure S4 MicroRNA scope staining of scrambled miRNA and IHC staining of IgG as the negative control from two groups are shown. Scale bar=50μm.

Figure S5:

The results showed that there was no significant difference in the uptake of CXCR4-Exo and Ctrl-Exo by HBMSC, HGEC and HUVEC.

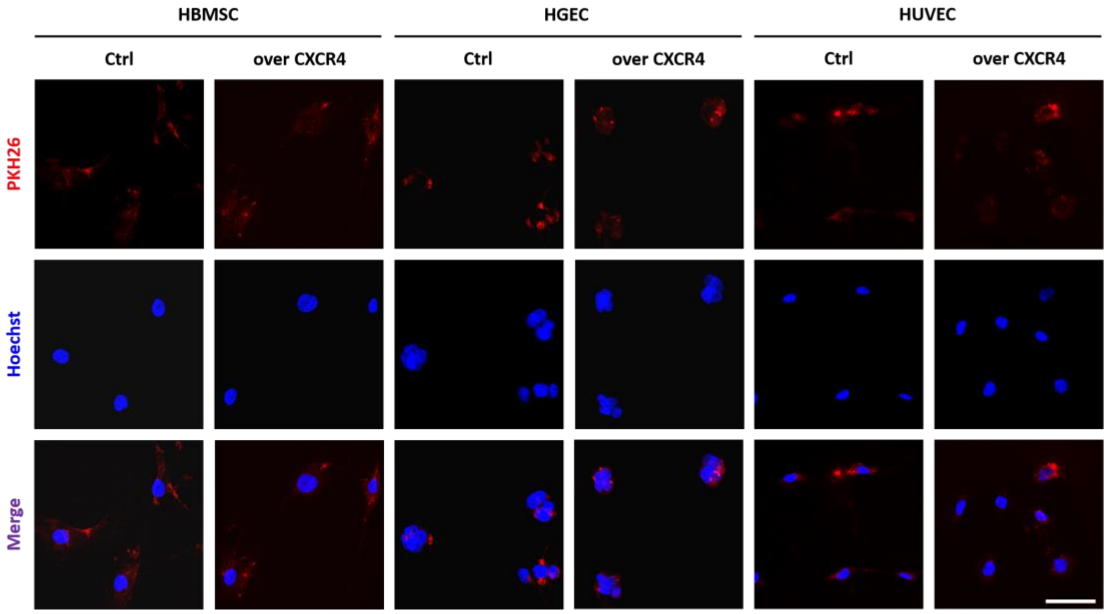

Figure S5 Representative IF images of exosome internalization from HBMSC, HGEC and HUVEC co-cultured with CXCR4-Exo and Crtl-Exo. Nuclei were stained with Hoechst. Scale bar = 50μm.

Figure S6:

Here showed the 3D reconstruction images of the maxillary second molar and alveolar bone of rats in four groups except Figure 6B.

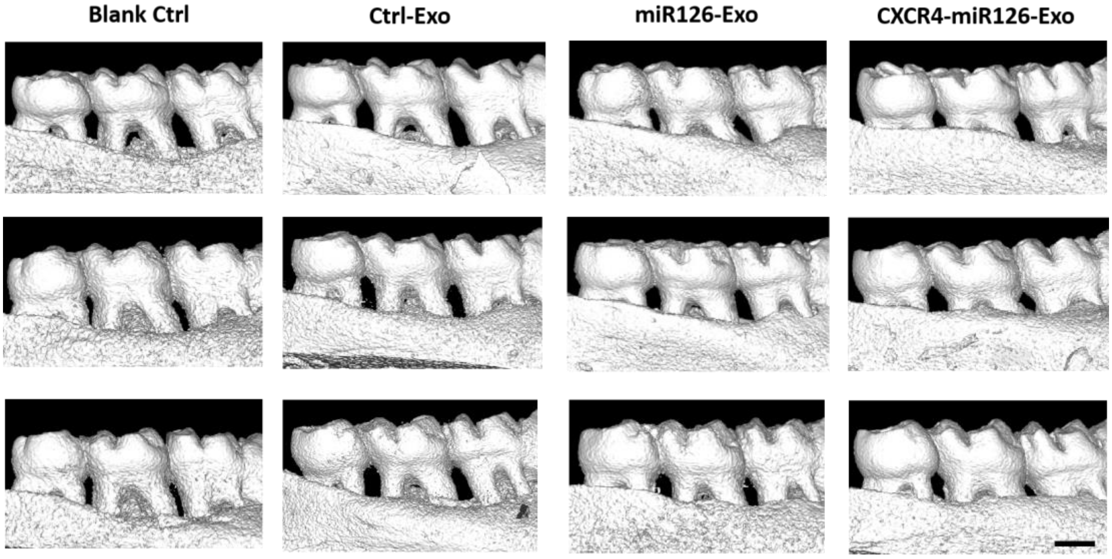

Figure S6 Representative images of a 3D reconstruction of the maxillary second molar and alveolar bone of the Blank Ctrl group, the Ctrl-Exo group, the miR126-Exo group and the CXCR4-miR126-Exo group. Scale bar=1mm.

Figure S7:

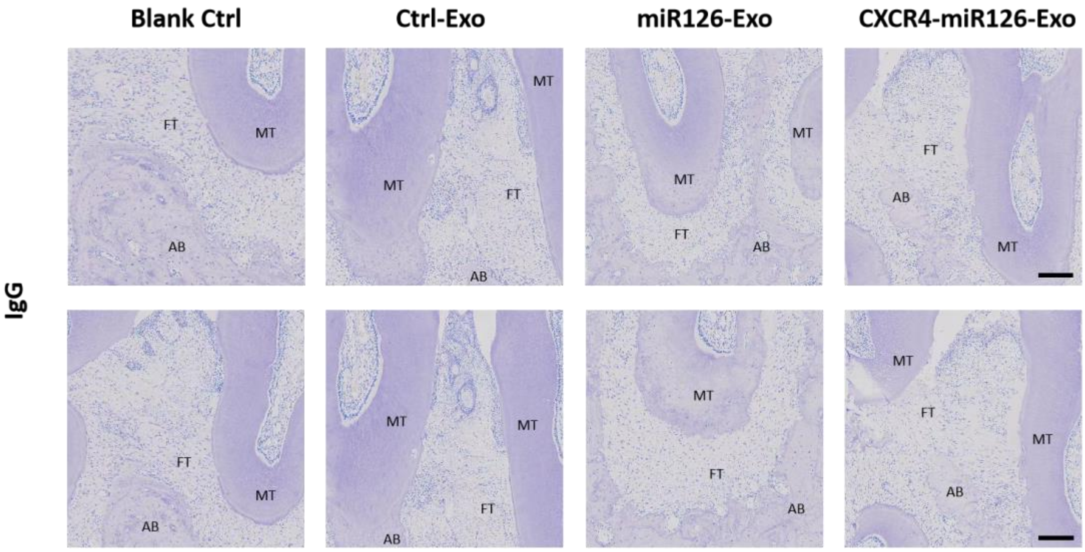

Figure S7 Representative immunohistochemical staining images of IgG as a negative control in each group. Scale bar=200μm. AB: alveolar bone; FT: fibrous tissues; MT: molar tissues.

Figure S8:

The expression of CD80 and CD86 in M0 and M1 macrophages were analyzed using the Cytoflex Flow Cytometry (Beckman Counlter, USA) and the CytExpert software (Beckman Counlter, USA). The cells were blocked with 3μL Human Trustain FcX (Fc receptor Blocking Solution, BioLegend, USA) for 10 min at room temperature, and stained with 3μL APC anti-human CD80 antibody (BioLegend, USA) and 3μL PE anti-human CD86 antibody (BioLegend, USA) in the dark for 30 min at 4°C.

Flow cytometry data showed that the M0 macrophages exhibited low expression levels of CD80 and CD86, with double-positive cells accounting for 15.16%, while the M1 macrophages exhibited high expression levels of CD80 and CD86, with double-positive cells accounting for 83.55%.

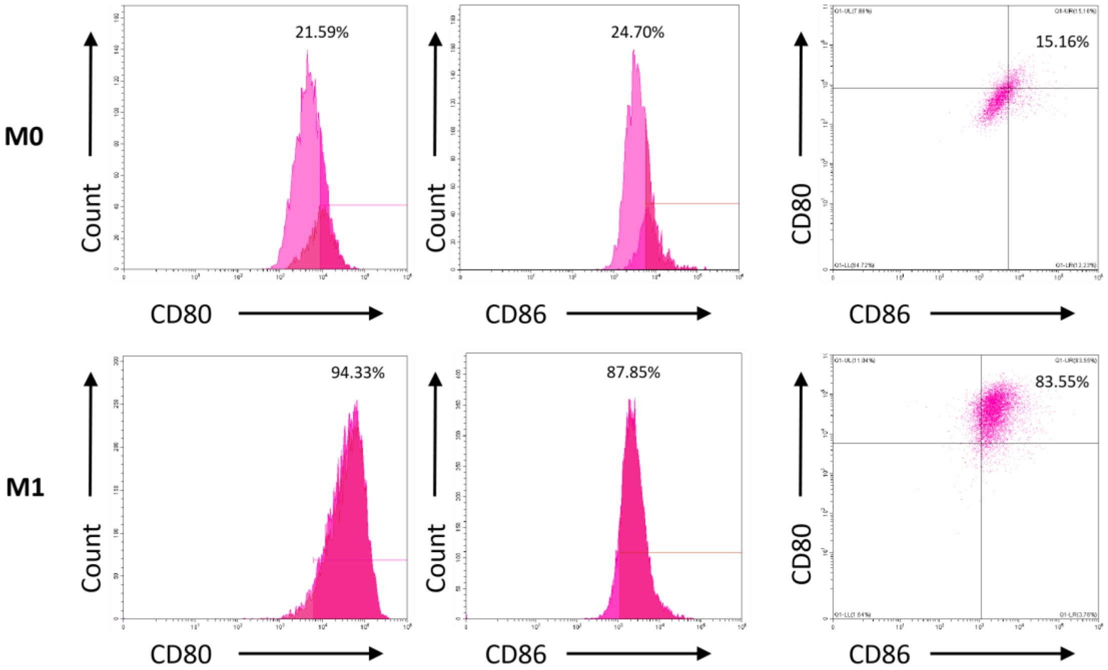

Figure S8　Flow cytometry analysis of CD80 and CD86 expression on M0 and M1 macrophages.

Figure S9:

It was observed by the DsRed fluorescence that miR-126 had high transfection efficiency in the CXCR4-overexpressing 293T/17 cells.

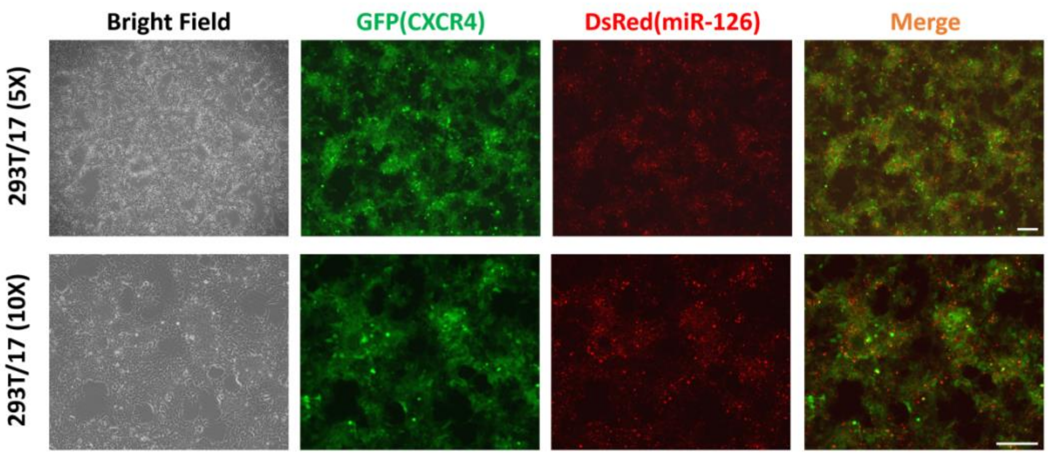

Figure S9 Transfection efficiency of miR-126 in the CXCR4-overexpressing 293T/17 cells. Scale bar=200μm.

Figure S10:

The GFP fluorescence revealed that both over-CXCR4 group and negative control group had high transfection efficiency in the 293T/17 cells.

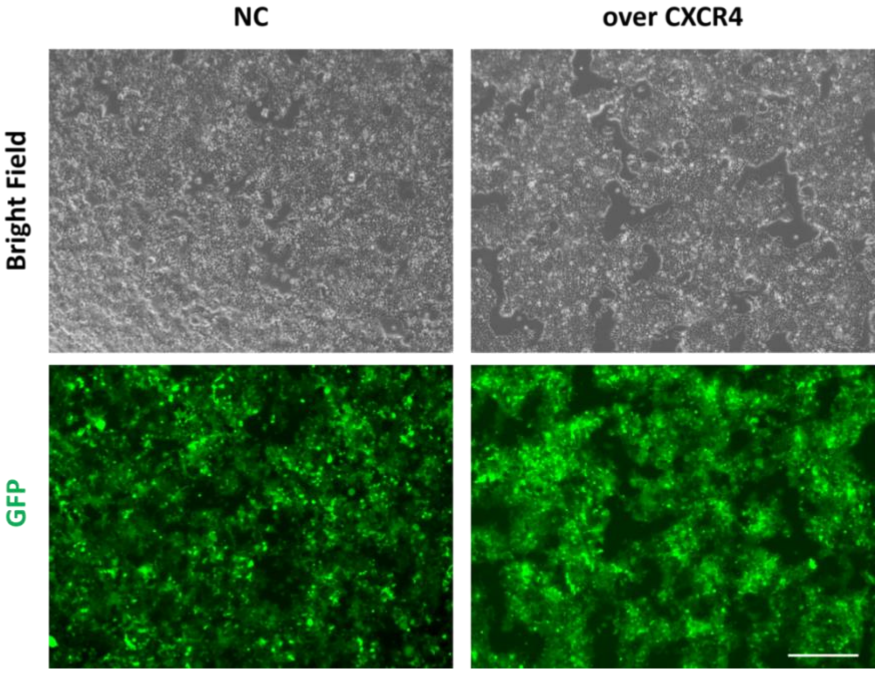

Figure S10 Transfection efficiency of CXCR4 in the 293T/17 cells. Scale bar=500μm.

Figure S11:

The results showed the successful construction of the periodontitis rat model. (A) The microCT analysis indicated the significant alveolar bone resorption in the maxillary second molars of the periodontitis rats. (B) Compared with the healthy control group, obvious ulceration of the gingival epithelium of the periodontitis group was observed in the H&E staining. (C) The immunohistochemical staining of CD68 showed only a small number of positive cells were found in the healthy control group, while a large number of positive cells were presented in the periodontitis group.

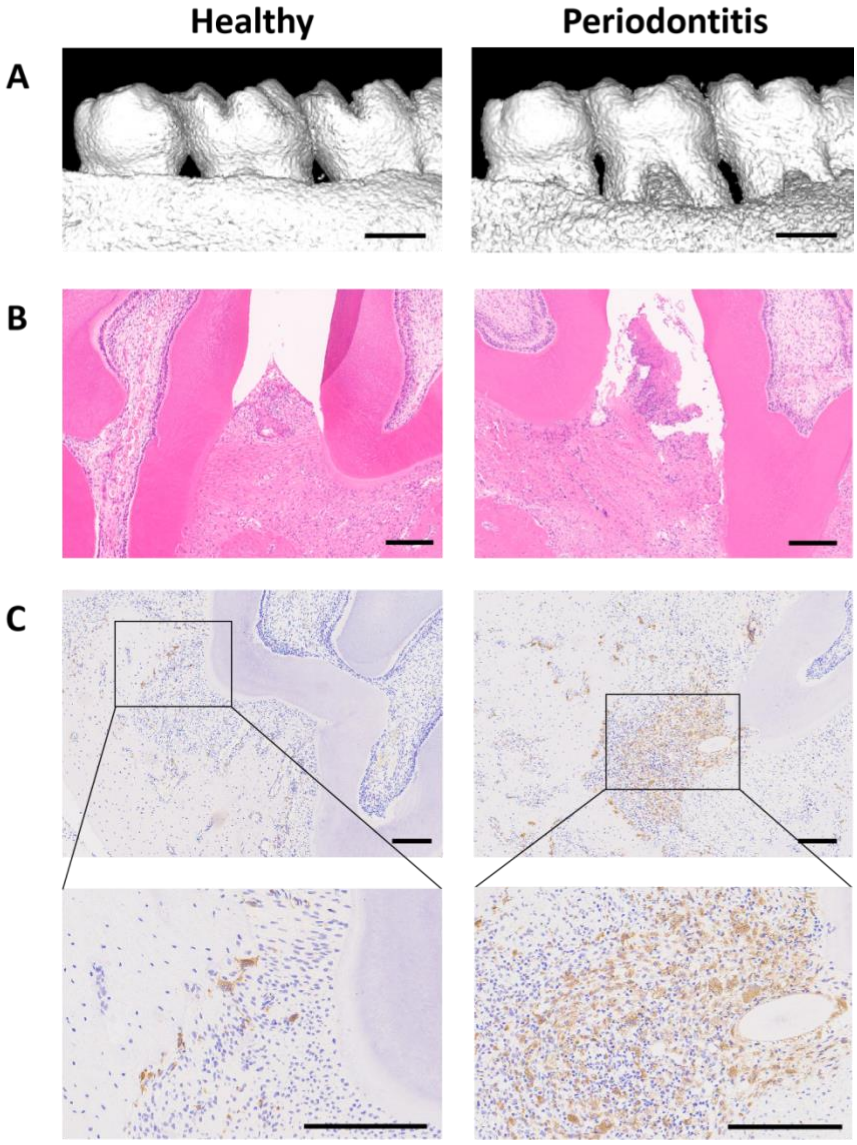

Figure S11 **(A)** Representative images of 3D reconstruction of the maxillary second molars and alveolar bone of the healthy control group and the periodontitis group. Scale bar=1mm. **(B)** The H&E staining of the periodontium in two groups. Scale bar=200μm. **(C)** Representative immunohistochemical staining images of CD68 in two groups. Scale bar=200μm.
